# Reduction of Mycotoxins during Fermentation of Whole Grain Sorghum to Whole Grain *Ting* (a Southern African Food)

**DOI:** 10.3390/toxins11030180

**Published:** 2019-03-25

**Authors:** Oluwafemi Ayodeji Adebo, Eugenie Kayitesi, Patrick Berka Njobeh

**Affiliations:** Department of Biotechnology and Food Technology, University of Johannesburg, P.O. Box 17011, Doornfontein 2028, South Africa; eugeniek@uj.ac.za (E.K.); pnjobeh@uj.ac.za (P.B.N.)

**Keywords:** sorghum, *ting*, fermentation, *Lactobacillus fermentum*, mycotoxins, food safety

## Abstract

Mycotoxins are fungal secondary metabolites that pose health risks to exposed individuals, requiring necessary measures to reduce them. Using liquid chromatography-tandem mass spectrometry (LC-MS/MS), mycotoxins were quantified in whole grain sorghum and *ting* subsequently derived from two sorghum varieties (high and low tannin). The whole grain (WG) *ting* samples were obtained by fermenting sorghum with *Lactobacillus fermentum* strains (FUA 3165 and FUA 3321). Naturally (spontaneously) fermented WG-*ting* under the same conditions were equally analysed. Among the mycotoxins investigated, fumonisin B_1_ (FB_1_), B_2_ (FB_2_), B_3_ (FB_3_), T-2 toxin (T-2), zearalenone (ZEA), alpha-zearalenol (α-ZOL) and beta-zearalenol (β-ZOL) were detected in sorghum. Results obtained showed that mycotoxin concentrations significantly (*p* ≤ 0.05) reduced after fermentation. In particular, *L. fermentum* FUA 3321 showed the capability to significantly (*p* ≤ 0.05) reduce all the mycotoxins by 98% for FB_1_, 84% for T-2 and up to 82% for α-ZOL, compared to raw low tannin sorghum. Fermenting with the *L. fermentum* strains showed potential to effectively reduce mycotoxin contamination in whole grain *ting*. Thus, we recommended *L. fermentum* FUA 3321 in particular to be used as a potential starter culture in sorghum fermentation.

## 1. Introduction

Mycotoxins are deleterious and of global public health concern, with numerous reported adverse health and economic effects [1,2]. These naturally occurring toxic compounds are frequent contaminants of agricultural commodities, significantly contributing to food losses and exposing the world’s population to a high health risk. In particular, chronic health risks arising from mycotoxin contamination are a main concern for the South African population, since major staple foods are highly contaminated by mycotoxins [1,3,4]. The climatic and environmental conditions in South Africa are characterised by temperate and humid conditions, erratic rainfall and frequent drought episodes, which are ideal conditions for mycotoxigenic fungal growth and proliferation. A detailed review by Misihairabgwi et al. [3] highlighted incidences of these mycotoxins in Southern African foods, as well as the link between mycotoxin contamination and aggravating health-related challenges including infant malnutrition, kwashiorkor and marasmus. Fumonisins (FBs) have been of significant prominence in South Africa since they were first discovered in 1988 [5], and are implicated in outbreaks of equine leukoencephalomalacia, porcine pulmonary edema, neural tube defects and esophageal cancer [5,6,7,8].

Cereals have been identified as a major route of dietary exposure to mycotoxins, which is of global concern, especially when these mycotoxins are carried over to subsequent products derived from these cereals [9,10,11]. Sorghum is an important cereal crop that is prevalent in Africa [12]. However, sorghum, like other cereal crops, is susceptible to fungal proliferation during cultivation, harvest, storage and processing. This colonisation of sorghum by toxigenic fungi could be accompanied by the production of secondary metabolites including mycotoxins [9,10], further aggravated by the favourable tropical climatic conditions that are prevalent in Africa. When ingested, these mycotoxins can cause harmful health effects including cancer and in extreme cases may lead to death [11]. Notable mycotoxins in sorghum include aflatoxins (AFs), fumonisins (FBs), zearalenone (ZEA), deoxynivalenol (DON) and ochratoxin A (OTA) [9,10,11,13,14]. Except for AFs, limited reports exist on mycotoxin occurrence in dietary products in southern African countries [15].

Fermentation is a traditional, age-old technique of transforming sorghum grains, or any other grain, into diverse forms of food that constitute the daily diets of most African populations. This processing technique has been well documented to improve shelf life, nutrient bioavailability and health benefits [16,17]. Porridges are staple foods in Southern African cuisine. An indigenous fermented sorghum-based porridge known as *ting* is commonly consumed as *bogobe* or *motogo* in South Africa, Botswana and other neighbouring Southern African countries [18,19,20]. The product is frequently fed to infants and regularly consumed by adults.

Considering the deleterious effects of mycotoxins, there is a need to explore viable, safe and practicable strategies that can mitigate their presence in foods. Several approaches of pre- and postharvest measures have not necessarily met the desired efficacy, safety levels and nutrient retention. Microbial decontamination provides a viable alternative for the possible removal of these toxic substances in foods under mild conditions, limiting significant losses in the aesthetic quality of food products [21,22,23,24]. Available reports in the literature have suggested fermentation, particularly with lactic acid bacteria (LAB) starter cultures, as an effective and promising technique for reducing the presence of mycotoxins while improving food composition, conferring preservative effects and retaining nutritive value [25,26,27,28,29,30].

With these naturally occurring toxic compounds that frequently contaminate agricultural commodities posing a hazardous risk to humans and animals, this study explored the reduction of these toxins through natural and lactic acid bacteria (LAB) fermentation during *ting* production from whole grain (WG) sorghum.

## 2. Results and Discussion

### 2.1. Presence of Mycotoxins

As observed in Table 1, the method validation results fulfilled the generally acceptable parameters [31]. Of the 14 mycotoxins investigated in this study (Table 1), seven including the fumonisins (FB_1_, FB_2_ and FB_3_), T-2 toxin (T-2), ZEA, alpha-zearalenol (α-ZOL) and beta-zearalenol (β-ZOL) were detected in raw WG-sorghum grain and subsequent WG-*ting* samples (Table 2). It was observed that the raw low tannin (LT)-sorghum samples generally had higher levels of mycotoxins than the high tannin (HT)-sorghum samples (Table 2). While LT-sorghum samples had FB_1_, FB_2_ and FB_3_ levels of 163, 12 and 400 μg/kg, respectively, the HT-sorghum samples contained neither FB_1_ nor FB_2_ though the same sample had FB_3_ at a level of 148 μg/kg. Higher mycotoxin levels in LT-sorghum samples when compared to their HT counterparts were equally recorded for T-2, α-ZOL and β-ZOL (Table 2). It was established in previous studies that there were higher concentrations of bioactive compounds (such as polyphenols, flavonoids and tannins) in HT-sorghum as compared to LT-sorghum [18,20,30]. Thus, we can reinforce the hypothesis that these compounds may have contributed to the inhibition of mycotoxigenic fungi and microbial action in addition to limiting attendant mycotoxin production, as demonstrated in other studies [32,33].

The levels of mycotoxins in the WG-sorghum samples recorded in this study are below the regulatory recommended mycotoxin limits in Southern Africa and the European Union (EU) [31,34,35], indicating that the LT- and HT-sorghum samples are quite “safe” for intended consumers. The relatively low mycotoxin levels observed in this study compared to other available studies in the literature on sorghum [10,11,13,14,27,36] could be the result of effective agricultural practices during its production, which might have limited the initial fungal contamination of the sorghum grains.

### 2.2. Mycotoxin Reduction

**Figure 1:**
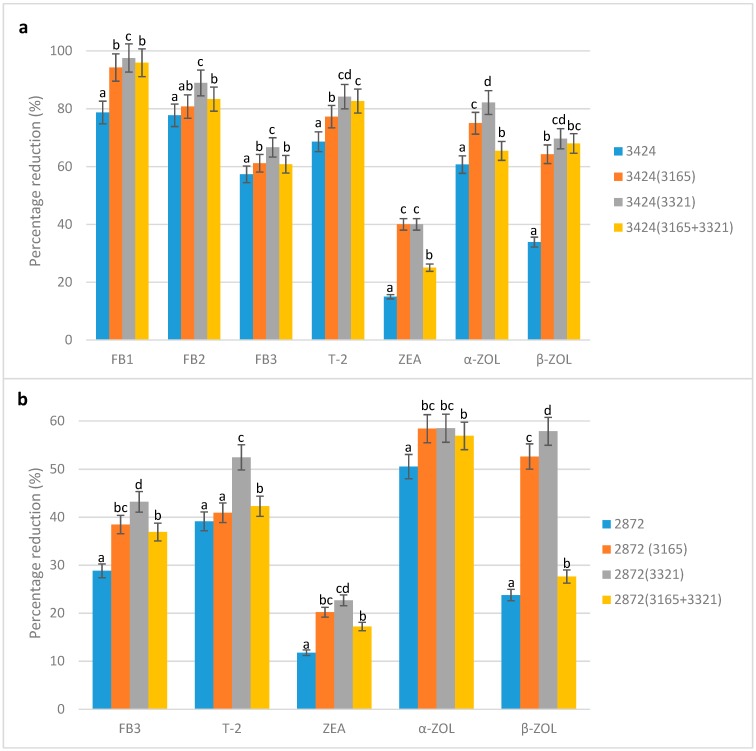
Reduction of mycotoxin levels in *ting* from whole grain sorghum. (**a**) *Ting* samples obtained from the LT-sorghum type; (**b**) *Ting* samples obtained from the HT-sorghum type. 2872—naturally fermented *ting* from HT-sorghum; 3424—naturally fermented *ting* from LT-sorghum type; (3165)—fermentation with *L. fermentum* FUA 3165; (3321)—fermentation with *L. fermentum* FUA 3321; (3165 + 3321)—fermentation with *L. fermentum* FUA 3165 and *L. fermentum* FUA 3321.

The presence of all the detected mycotoxins was significantly (*p* ≤ 0.05) reduced after the fermentation of WG-sorghum to WG-*ting* (Table 2). Residual mycotoxin levels in LT-*ting* samples were FB_1_ (4–34.68 μg/kg), FB_2_ (1.33–2.67 μg/kg), FB_3_ (133.33–170.67 μg/kg), T-2 (1.17–2.32 μg/kg), ZEA (4.00–5.67 μg/kg), α-ZOL (5–11 μg/kg) and β-ZOL (11.83–24.67 μg/kg). For HT-*ting* samples, residual mycotoxin levels ranged between 84.06 and 105.33, between 3.17 and 4.06, between 4.67 and 5.33, between 8.67 and 10.33 and between 10.67 and 19.31 μg/kg for FB_3_, T-2, ZEA, α-ZOL and β-ZOL, respectively (Table 2). Although the sorghum varieties were fermented in different conditions, i.e., 24 h at 34 °C (LT-sorghum type) and 72 h at 28 °C (HT-sorghum type), fermentation with *L. fermentum* strains both singly and in combination was observed to be more effective at reducing mycotoxins in subsequent WG-*ting* samples derived from them (Figure 1a,b).

Natural fermentation of the LT-*ting* samples resulted in 79, 78, 57, 69, 15, 61 and 34% reductions in FB_1_, FB_2_, FB_3_, T-2, ZEA, α-ZOL and β-ZOL, respectively (Figure 1a). Lesser reduction levels were, however, observed in the HT-*ting* samples (Figure 1b). Percentage reduction of the mycotoxins after fermentation with *L. fermentum* strains was more pronounced and above 60% in the LT-*ting* samples (Figure 1a) and above 25% in the HT-*ting* samples. These reductions were relatively low in naturally (spontaneously) fermented WG-*ting* samples obtained from both LT- and HT-sorghum samples (Figure 1a,b). Although the fermentation time for HT-*ting* samples was longer, the percentage of mycotoxin reduction in LT-*ting* samples was higher, implying that initial substrate composition could have influenced the extent of mycotoxin reduction. This is equally reflected in the higher microbial population observed in the LT-*ting* samples compared to their HT-counterpart in our earlier study [18]. The high percentage FB reduction of over 90% in all of the LAB-fermented LT-*ting* samples recorded in this study (Figure 1a) is worthy of note. Similar findings have been reported in other African fermented foods such as fermented maize gruel, *ogi* and *mahewu* [28,37,38,39]. This is particularly important, considering the deleterious effect of these FBs and their harmful effects to inhabitants of southern Africa and the world at large.

The composition of microbiota largely influences the products of microbial metabolism during fermentation. As observed in our earlier study, relatively low pH values, higher corresponding titratable acidity (TTA) values and significantly (*p* ≤ 0.05) higher microbial loads were recorded during *ting* production with the *L. fermentum* strains [18]. Relatively low pH, high alcohol content, lactic acid and increased production of other relevant metabolites during the fermentation with LABs could have instigated a better mycotoxin reduction during LAB fermentation. Consequent reduction could be attributed to a possible breakdown and/or degradation of mycotoxins to less toxic products by the fermenting microbiota. Fermentation has been identified as an effective process to reduce mycotoxins due to their breakdown by endogenous enzymes and compounds secreted and released into the food matrix by these fermenting organisms [21,29]. The production of bacteriocins as well as antagonistic and proteinaceous compounds might have also contributed to this observation. As observed in our previous study [18], the production of such compounds could have also resulted in the reduction of fungal load with the LAB-fermented *ting* samples compared to the spontaneously fermented ones.

The significantly (*p* ≤ 0.05) lower residual mycotoxin levels and corresponding higher percentages of mycotoxin reduction in WG-*ting* samples obtained using *L. fermentum* FUA 3321 (singly) demonstrates the highest mycotoxin reduction of all the tested mycotoxins. In a study on FB_1_ and FB_2_ reduction by *Lactobacillus* strains, Zhao et al. [40] attributed their efficacy to an enhanced interaction between mycotoxins and the bacterial cell wall. Likewise, this interaction could be a reason for the effectiveness of *L. fermentum* FUA 3321 at reducing mycotoxins. Relatively low mycotoxin reduction in HT-*ting* samples (Figure 1b) compared to LT-*ting* samples (Figure 1a) and the generally higher reduction with LAB strains (Figure 1a,b) compared to natural fermentation could be attributed to accelerated fermentation and increased microbial action. Not only was *L. fermentum* FUA 3321 a better fermenting microorganism, as observed in an earlier study [18], a faster decontamination process and rapid metabolism of the strain could have contributed to this observation. Studies have shown that fermentative organisms such as LABs are capable of adsorbing mycotoxins from agricultural products onto their cell wall surface components, thereby effectively decontaminating food [41,42]. As indicated in previous studies [21,28,43], LABs can better detoxify mycotoxins to less toxic forms during cereal fermentation. It could thus be speculated that these toxins have been completely detoxified, hydrolysed and degraded to less toxic forms.

Although effective mycotoxin reduction in this study was highest for *L. fermentum* FUA 3321 alone, followed by *L. fermentum* FUA 3165 alone, the combination of *L. fermentum* FUA 3321 and 3165 and then natural fermentation (Figure 1a,b), this trend was distinctly different for T-2 (Table 2, Figure 1a,b). While *L. fermentum* FUA 3321 was still the most effective strain, a combination of *L. fermentum* FUA 3321 and 3165 proved more effective than *L. fermentum* FUA 3165 when used in isolation. It can thus be suggested that the synergistic effect of the mixed LABs on T-2 reduction was more effective in binary combination (*L. fermentum* FUA 3165 and *L. fermentum* FUA 3321) compared to a pure culture of *L. fermentum* FUA 3165. It could also be speculated that a single strain of *L. fermentum* FUA 3321 and the binary combination of the strains had relatively higher affinity for the 12,13-epoxy ring responsible for the toxicity of T-2 [44]. On the contrary, the lower percentage reduction of ZEA (Figure 1a,b) could rather be due to the reduced affinity of the strains to the lactone ring of the ZEA molecule.

While these mycotoxins are all known to pose significant threats and illicit toxic effects, their reduction levels vary as recorded in Table 2 and Figure 1. This variation in the extent of mycotoxin reduction can also be attributed to differences in their chemical structure, polarity, dissociation constant and initial mycotoxin levels/concentration, all of which contributed to the reduction levels obtained. Furthermore, several other compounds have the capability of reacting with the cell wall of fermenting strains, making them equal competitors for the mycotoxin detoxification process [45,46]. While all of this might have occurred during both natural and LAB fermentation processes of *ting*, a better interaction between the cell wall of *L. fermentum* FUA 3321 and the mycotoxins could have resulted in a significantly (*p* ≤ 0.05) higher percentage mycotoxin reduction observed. Although not investigated in this study, the size, shape, surface area and the surface charge of the *L. fermentum* FUA 3321 cells could have also contributed to this observation.

## 3. Conclusions

Fermentation significantly reduced the levels of mycotoxins in *ting* samples obtained from whole grain sorghum samples, especially with the use of lactic acid bacteria strains. Although both *L. fermentum* strains exhibited good mycotoxin reduction ability, the use of *L. fermentum* FUA 3321 resulted in a better mycotoxin reduction. It can thus be deduced that *L. fermentum* strains are promising and suitable starter cultures for dietary detoxification of mycotoxins in fermented food commodities. Further research is still needed to investigate the activity of these strains on other fermented foods and to clarify whether the mycotoxins apparently lost during WG-*ting* preparation are indeed destroyed, hydrolysed or bound to the food matrix to become non-recoverable. Additionally needed and equally important are toxicity studies to ascertain the toxic nature of the decontamination products thereof.

## 4. Materials and Methods

### 4.1. Raw Material and Sample Preparation

Sorghum (*Sorghum bicolor* L.) grain cultivars, i.e., high tannin (HT) and low tannin (LT) obtained and prepared as reported in Adebo et al. [20], were used. The tannin content (TNC) of both raw LT- and HT-sorghum types was investigated, with values recorded as 17.97 and 31.68 mg CE/g. As such, these were subsequently classified as LT- and HT-sorghum types, respectively [18].

### 4.2. Lactobacillus Strains

Two *L. fermentum* strains (*L. fermentum* FUA 3165 and *L. fermentum* FUA 3321) used were earlier isolated from *ting* [10] and prepared as described in [18].

### 4.3. Fermentation of Sorghum into WG-Ting

*Ting* was processed by mixing 50 g of WG-sorghum flour and sterile distilled water (40 °C) (1:1, *w*/*v*) [20]. Fermentation with the LAB strains was conducted as earlier described [18]. Naturally fermented WG-*ting* samples were also obtained by spontaneously fermenting sorghum under similar conditions (without the strains). All samples were freeze-dried at −55 °C for 24 h prior to analysis.

### 4.4. Mycotoxin Standards

Mycotoxin standards including AFB_1_, AFB_2_, AFG_1_, AFG_2_ and DON were purchased from Sigma Aldrich, Germany, while FB_1_, FB_2_, FB_3_, OTA, OTB, T-2, ZEA, α-ZOL and β-ZOL were obtained from the Council for Scientific and Industrial Research (CSIR), South Africa. They were all dissolved in LC-grade methanol (MeOH) and prepared at five different concentration levels ranging between 0.0002 and 0.2 μg/L (OTA, OTB), 0.001 and 1 μg/L (AFB_1_, AFB_2_, AFG_1_, AFG_2_), 0.0025 and 2.5 μg/L (FB_1_, FB_2_, FB_3_, ZEA, α-ZOL, β-ZOL) and 0.05 and 5 μg/L (DON, T-2). This was subsequently used to obtain a blank (neat solvent) calibration curve for initial mycotoxin quantification.

### 4.5. Mycotoxin Extraction

A modified Quick Easy Cheap Effective Rugged and Safe (QuEChERS) method earlier adapted by Oueslati et al. [36] and Motloung et al. [47] was followed for mycotoxin extraction. Freeze-dried raw sorghum and *ting* samples were finely milled and homogenised. Then, 1 g was weighed into an extraction tube containing 5 mL of distilled water, vortexed and left for 30 min. Thereafter, 5 mL (acetonitrile (ACN)/1% formic acid, *v*/*v*) extraction solvent was added and sonicated for 20 min. NaCl (0.5 g) and 2 g of MgSO_4_ anhydrous salt were added; the tube was capped and briefly shaken to avoid agglomeration. The tubes were vortexed, centrifuged for 15 min at 4000 × *g* and the supernatant layer was filtered (0.22 µm, Millipore, Bedford, MA, USA) into vials for LC-MS/MS analysis.

### 4.6. Mycotoxin Analysis

For the quantification of the mycotoxins, triplicates of each extract were analysed in multiple reaction monitoring (MRM) mode by injecting 10 µL of each into the LC-MS/MS system that consisted of a UPLC instrument (Shimadzu Kyoto, Japan) equipped with an auto-sampler (SIL-30 AC, Nexera, Kyoto, Japan), communication bus module (CBM-20A), column oven (CTO-30A), degassing unit (DGU-20A5R) and a liquid chromatograph (LC-30AD) interfaced with a triple quadrupole mass spectrometer (LC-MS-8030). A Raptor C_18_ column (2.7 µm particle size × 100 mm length × 2.1 mm ID, Restek, Bellefonte, PA, USA) was used and the analysis was performed at a constant flow rate of 0.2 mL/min. The mobile phases, solvents A and B, consisted of 0.1% formic acid in water and 0.1% formic acid in ACN:MeOH (50:50, *v*/*v*), respectively. The solvent gradients were 10% B for 0.1 min, ramped to 95% B over 8.4 min, held at 95% B for 3 min and then 10% B for 1 min, after which the column was re-equilibrated at this condition for 4.5 min. The column temperature was maintained at 40 °C throughout the chromatographic runs and MS data were acquired in a positive mode. The interface nebulising gas flow rate was 3 L/min, the DL temperature was 250 °C, the heat block temperature was 400 °C and the drying gas flow rate was 15 L/min. Other optimal LC-MS/MS parameters are presented in Table 1.

Apparent recovery rates (ARR) were accessed by spiking 1 g of sorghum “blank samples” with known concentrations of mycotoxins in triplicates and maintaining them at room temperature overnight for solvent evaporation [48]. The mycotoxin was re-extracted and quantified as earlier described in this section. Matrix (sorghum)-matched calibration curves were also prepared from the sorghum matrix and subsequent results were based on the ARR obtained and quantification obtained through the matrix-matched calibration curve. The limits of detection (LOD) and quantification (LOQ) were determined using the method described by Wenzl et al. [49]. Percentage mycotoxin reduction after fermentation was calculated as [(A − B)/A] × 100, where A and B are the initial and final mycotoxin concentrations, respectively [50].

### 4.7. Statistical Analysis

All analyses were conducted in triplicate and the results presented represent the average of triplicate determinations. An analysis of variance (ANOVA) was performed using SPSS 22 (IBM, New York, NY, USA) [32] and mean values among treatments for each sample type were considered to differ significantly if *p* ≤ 0.05.

## Figures and Tables

**Table 1:** Identity and characteristics of the mycotoxins investigated by liquid chromatography-tandem mass spectrometry (LC-MS/MS).

R_t_ (min)	Mycotoxin Standard	MW	Parent Ion *m*/*z* (Precursor)	MS/MS Fragments	CE	R^2^ (Neat Solvent)	R^2^ (Sorghum Mix)	ARR (%)	LOD (µg/kg)	LOQ (µg/kg)
2.78	DON	296	297.10	231, 249	12	0.989	0.981	89	0.11	1.74
6.87	AFG_2_	330	331.00	245, 313	32	0.999	0.992	82	0.01	0.04
7.12	AFG_1_	328	329.00	243, 311	28	0.999	0.990	85	0.01	0.04
7.30	AFB_2_	314	315.00	259, 287	31	0.999	0.989	91	0.59	1.98
7.48	AFB_1_	312	313.00	241, 285	24	0.999	0.997	90	0.01	0.04
7.58	FB_1_	721	722.40	352, 334	42	0.997	0.996	98	0.34	0.87
8.03	β-ZOL	322	323.00	277, 305	11	0.998	0.998	90	0.21	0.71
8.20	FB_2_	705	706.10	336, 318	38	0.999	0.996	99	0.41	0.93
8.25	FB_3_	705	706.30	336, 354	35	0.999	0.997	94	1.02	4.11
8.28	OTB	369	370.10	205, 324	14	0.998	0.991	94	0.06	0.19
8.38	α-ZOL	322	323.10	277, 305	9	0.999	0.993	87	0.65	2.17
8.53	T-2	466	467.20	245, 305	11	0.999	0.997	96	0.39	0.90
8.74	ZEA	318	319.10	185, 187	21	0.999	0.991	92	0.03	0.11
8.78	OTA	403	404.00	239, 221	38	0.999	0.993	86	0.04	0.14

ARR—apparent recovery rate; CE—collision energy; R_t_—retention time; LOD—limit of detection; LOQ—limit of quantification; MW—molecular weight; *m*/*z*—mass-to-charge ratio; MS/MS—tandem mass spectrometry; R^2^—coefficient of regression; AFB_1_—aflatoxin B_1_; AFB_2_—aflatoxin B_2_; AFG_1_—aflatoxin G_1_; AFG_2_—aflatoxin G_2_; DON—deoxynivalenol; FB_1_—fumonisin B_1_; FB_2_—fumonisin B_2_; FB_3_—fumonisin B_3_; OTA—ochratoxin A; OTB—ochratoxin B; T-2—T-2 toxin; ZEA—zearalenone; α-ZOL—alpha-zearalenol; and β-ZOL—beta-zearalenol.

**Table 2:** Quantification of mycotoxins (µg/kg) in sorghum and after fermentation to whole grain *ting*.

	FB_1_	FB_2_	FB_3_	T-2	ZEA	α-ZOL	β-ZOL
**LT-sorghum**							
Raw LT-sorghum	162.67 ^d^ ± 3.90	12.00 ^b^ ± 0.99	400.00 ^d^ ± 2.98	7.39 ^b^ ± 1.20	6.67 ^c^ ± 0.50	28.00 ^d^ ± 0.45	37.33 ^d^ ± 0.53
3424	34.68 ^c^ ± 5.58	2.67 ^a^ ± 0.45	170.67 ^c^ ± 2.35	2.32 ^a^ ± 0.74	5.67 ^b^ ± 0.28	11.00 ^c^ ± 0.99	24.67 ^c^ ± 0.49
3424 + 3165	9.33 ^b^ ± 1.40	2.31 ^a^ ± 0.98	155.33 ^b^ ± 1.45	1.68 ^a^ ± 0.78	4.00 ^a^ ± 0.82	7.00 ^b^ ± 0.97	13.33 ^b^ ± 0.75
3424 + 3321	4.00 ^a^ ± 2.63	1.33 ^a^ ± 1.06	133.33 ^a^ ± 2.19	1.17 ^a^ ± 0.63	4.00 ^a^ ± 0.05	5.00 ^a^ ± 0.92	11.83 ^a^ ± 0.63
3424 (3165 + 3321)	6.67 ^ab^ ± 2.10	2.00 ^a^ ± 0.92	156.67 ^b^ ± 2.09	1.28 ^a^ ± 0.75	5.00 ^b^ ± 0.06	9.67 ^c^ ± 0.81	11.94 ^a^ ± 0.07
**HT-sorghum**							
Raw HT-sorghum	–	–	148.00 ^d^ ± 1.93	6.67 ^b^ ± 1.00	6.04 ^b^ ± 0.13	20.89 ^c^ ± 0.82	25.33 ^c^ ± 0.44
2872	–	–	105.33 ^c^ ± 1.80	4.06 ^a^ ± 0.06	5.33 ^ab^ ± 0.43	10.33 ^b^ ± 0.44	19.31 ^b^ ± 0.44
2872 + 3165	–	–	91.07 ^b^ ± 1.74	3.94 ^a^ ± 0.85	4.82 ^a^ ± 0.1	8.69 ^a^ ± 0.21	12.00 ^a^ ± 0.87
2872 + 3321	–	–	84.06 ^a^ ± 2.29	3.17 ^a^ ± 0.17	4.67 ^a^ ± 0.3	8.67 ^a^ ± 0.51	10.67 ^a^ ± 0.46
2872 (3165 + 3321)	–	–	93.38 ^b^ ± 1.82	3.85 ^a^ ± 0.33	5.00 ^a^ ± 0.71	9.00 ^a^ ± 0.23	18.33 ^b^ ± 1.21

HT—high tannin; LT—low tannin; 2872—naturally fermented *ting* from HT-sorghum type; (3165)—fermentation with *Lactobacillus fermentum* FUA 3165; (3321)—fermentation with *L. fermentum* FUA 3321; (3165 + 3321)—fermentation with *L. fermentum* FUA 3165 and *L. fermentum* FUA 3321; 3424—naturally fermented *ting* from LT-sorghum type. Each value is a mean ± standard deviation of triplicates. ^a,b,c,d^ No common letters within a column under each sample type significantly (*p* ≤ 0.05) differ. FB_1_—fumonisin B_1_, FB_2_—fumonisin B_2_; FB_3_—fumonisin B_3_, T-2—T-2 toxin; ZEA—zearalenone; α-ZOL—α-zearalenol and β-ZOL—β-zearalenol.
